# Hepatitis B Birth Dose among Children in District 2 Hospital, Ho Chi Minh City, Vietnam: Prevalence and Associated Factors

**DOI:** 10.1155/2020/5680154

**Published:** 2020-07-15

**Authors:** Giao Huynh, Thanh Binh Nguyen, Ngoc Nga Cao, Minh Hoang Phan, Thi Bich Hop Dang, Thi Ngoc Han Nguyen

**Affiliations:** ^1^Faculty of Public Health, University of Medicine and Pharmacy at Ho Chi Minh City, Ho Chi Minh City, Vietnam; ^2^Faculty of Medicine, Tra Vinh University, Tra Vinh Province, Vietnam; ^3^Department of Infectious Diseases, University of Medicine and Pharmacy at Ho Chi Minh City, Ho Chi Minh City, Vietnam; ^4^Department of Scientific Research, District 2 Hospital, Ho Chi Minh City, Vietnam; ^5^Department of Disease Control and HIV/AIDS, Tan Hong District Health Center, Tan Hong District, Dong Thap Province, Vietnam; ^6^Infection Control Department, University Medical Center Ho Chi Minh City, Ho Chi Minh City, Vietnam

## Abstract

**Background:**

A birth dose of the hepatitis B vaccine will prevent most perinatally acquired infections and offers early protection from horizontal transmission. This article assessed the prevalence of the hepatitis B birth dose and associated factors among children in the District 2 Hospital.

**Methods:**

A prospective cross-sectional study between June and December 2017 recruited parents/caregivers of children aged 12–59 months who were randomly selected at the vaccination department in the District 2 Hospital. The structured questionnaire applied was to collect the characteristics of participants and check the vaccination schedule. The birth dose was defined as the hepatitis B vaccine, which was given to children within 24 hours after birth. Additionally, a semistructured questionnaire was used for interviews and focus group discussions (FGDs) to assess the risk perceptions and barriers to vaccination.

**Results:**

A total of 292 parents/caregivers had a mean age of 32.7 ± 6.8 years; among them, 88.7% were females. Their children had a mean age of 30.3 ± 13.9 months and 71.6% of these children received the hepatitis B birth dose, which correlated with the age of gestation (*P* < 0.05). In-depth interviews and FGDs found that most participants did not know that hepatitis B could be transmitted through childbirth, and barriers that affected the birth dose vaccine included children being sick, premature infants, or reason relating to physicians.

**Conclusions:**

The rate of hepatitis B birth dose was low, which resulted from associated factors such as premature birth, likely to be linked with false contraindications and beliefs that, potentially, the 2013 incident is still fresh in people's minds. Therefore, strategies to implement policies around the hepatitis B birth dose should be in line with current World Health Organization recommendations and strategies to modify current beliefs about vaccination.

## 1. Background

Hepatitis B virus (HBV) infection is considered a public health issue in Vietnam with 8.6 million people being HBV positive. The rate of infection in women is lower than men, being 8.8% and 12.3%, respectively [1]. In highly endemic areas, the majority of transmissions of HBV mainly occurs early in life, either from mother to child at birth (MTCT), or a lesser extent, from person to person during early childhood [1, 2]. HBV infection can lead to consequences of acute liver disease, cirrhosis, liver cancer, and death [2–4]. The probability of a person being chronically infected with HBV is conversely related to age. For children infected at birth, chronic HBV infection is recorded as 90%, if infected between 1 and 5 years of age, it is about 30%, and only 5–10% if infected after 5 years of age [5, 6]. If the mother is carrying the hepatitis B surface antigen (HBsAg), the risk of HBV infection among children vaccinated with the birth dose 7 days after birth was eight times higher than those vaccinated within the first three days. Therefore, the WHO recommends all children should receive a birth dose as soon as possible, preferably within 24 hours after birth [7]. In Vietnam, the hepatitis B vaccine has been utilized widely through the Expanded Program on Immunization (EPI) since 2002. Recommended vaccination schedules that include a first dose of the hepatitis B vaccine (birth dose) is to be given to infants within 24 hours after birth and followed by three additional doses of the pentavalent vaccine (diphtheria, pertussis, tetanus, hepatitis B, and Hib), which are recommended at 2, 3, and 4 months, that will protect from vertical and horizontal transmissions [8]. The HBV birth dose is a monovalent vaccine containing surface proteins of the hepatitis B virus absorbed by aluminum hydroxide or monophosphoryl lipid A adjuvant. Furthermore, the HBV birth dose was categorized as “timely” or “delayed” when offered within 24 hours after birth or between the second and seventh days [9]. From the hepatitis B vaccine birth dose introduction, the rate of vaccination coverage increased from 65% in 2006 to 75% in 2012. However, there was a dramatic decline to 55% in 2013 and 2014, likely as a result of a media report that started a chain of events that resulted in people being scared to vaccinate (health workers and/or caregivers) [10]. As a result, the effect of decreasing coverage resulted in around 130,675 new chronic HBV infections in children who were born in 2013 [11]. Although the events can be purely coincidental, the fear about vaccine safety widened and reduced in vaccine demand. Some previous studies in Vietnam showed that the low rate of HBV birth dose coverage was only 46.6% and 62.8% [9, 12]. The District 2 Hospital is one of the largest hospitals in Ho Chi Minh City, with specialist services, so that the response to major medical issues is available to all patients. There were many thousands of deliveries and vaccinations every year. Using this facility, this study aims to assess the prevalence of hepatitis B vaccine birth dose and associated factors among children aged 12–59 months in the District 2 Hospital.

## 2. Methods

### 2.1. Study Population

All parents/caregivers who directly take care of children and whose children are between 12 and 59 months of age, who had the vaccination cards, were eligible. A cross-sectional study was performed between June and December 2017. Systematic random sampling was based on the daily registered list to select children at the vaccination department. We selected every 3^rd^ child for sampling (*k* = 3). After that, a purposeful sampling strategy was carried out on a total of 30 parents/caregivers whose children did not receive the birth dose within 24 hours. The participants were invited to an interview or focus group discussion (FGDs).

### 2.2. Data Collection Procedures

The structured questionnaire included two sections. The first depicted the demographics of the parents/caregivers, such as age, gender, residence location, occupation, education, and information about hepatitis B vaccination, and the demographics of children included age, gender, birth weight, place of birth, and the period of delivery and birth order of the child in the family. The second assessed the hepatitis B birth dose vaccination by checking their children's vaccination records (paper records). If no vaccination card was available, the next child on the list was contacted. Additionally, a sample of 30 parents/caregivers, whose children did not receive the hepatitis B birth dose within 24 hours, undertook an in-depth interview or FGDs. A semistructured questionnaire designed for our in-depth interview questionnaire and FGDs was used in assessing risk perceptions (risk of getting the disease and transmission) and to identify barriers to receiving the vaccination (reasons that children did not receive the birth dose on time or difficulties in getting the hepatitis B vaccine) [13]. It took between 15 and 30 minutes per interview, and it took approximately 60–90 minutes per FGDs, comprising a total of 15 in-depth interviews (fifteen parents/caregivers) and 3 FGDs comprising of 5 parents/caregivers in each. Parents/caregivers could either finish the in-depth interviews or take part in the FGDs but not in both. The interviews and FGDs were recorded, fully duplicated, and verified. All were held at the District 2 Hospital.

### 2.3. Variable Definitions

We evaluated the hepatitis B vaccination based on the vaccination cards, whereby the period of time for the birth dose was calculated by the date of vaccination minus the date of birth; however, if the period of time falls within one day (24 hours), it is considered as a timely birth dose, and the reverse applied for vaccinations that were defined as delayed. The children were recorded as “complete and timely” when they were vaccinated with the timely birth dose, followed by the three additional doses of pentavalent vaccine, recommended at 2, 3, and 4 months [8].

### 2.4. Method of Analysis

We analyzed participants' demographics and the prevalence of the birth dose using Stata 13.0 software. Descriptive analysis was described as frequency, percentage, and mean scores. The chi-square or Fisher's exact test and the *t*-test were used to analyze the relationship between the dependent (demographic characteristics of participants) and independent variables (the birth dose of children). Logistic regression was performed in the multivariate analysis. All the significant differences of variables were considered if the *P* value showed <0.05. While data from the transcripts were coded using NVivo10, which was used to record our themes, tally code frequencies, and trace code relationships. We used a constant comparison method, by which we tested and applied our coding categories to each new transcript.

### 2.5. Ethics Approval and Consent to Participate

All participants agreed and signed informed consent forms before taking part in our study and were able to stop participating at any time. Also, they were assured that their responses would be kept confidential, and transcripts were anonymous. The proposal was approved by the Ethics Council of the University of Medicine and Pharmacy at Ho Chi Minh City (protocol number 196-UMP-BOARD).

## 3. Results

### 3.1. Demographic Characteristics of Respondents

A total of 688 eligible participants, with 292 (42.4%) of them agreeing to take part in completing the questionnaire. Demographics of the study population are presented in Table 1. A total of 292 parents/caregivers had a mean age of 32.7 ± 6.8 years old, 88.7% female, with the main occupation being government officer/staff (41.8%), and the highest education level recorded as the high school level (73.6%). In terms of children, the mean age was 30.3 ± 13.9 months old with slightly more males than females. Most children (96.9% and 94.9%) had a birth weight of more than 2,500 grams and a gestation period of more than 37 weeks with almost one-third of children delivered by cesarean (36.6%). Most children were born after 2013 (89.1%).

### 3.2. The Prevalence of the Hepatitis B Birth Dose Vaccine and Associated Factors

There were 94.9% of children that received three doses of pentavalent vaccine, while only 71.6% of them got the birth dose within 24 hours, and children who had complete and timely hepatitis B vaccine, including the birth dose and three doses of pentavalent vaccine on time, were recorded as 71.2% (Table 2).

The comparisons of participants' characteristics in groups with and without the hepatitis B birth dose vaccine within 24 hours are presented in Table 3, in which having hepatitis B vaccination information and the age of gestation correlated with the percentage of the timely birth dose (*P* < 0.05). Adjusting for other variables, the gestation period of more than 37 weeks had a 1.2 times higher prevalence of receiving the birth dose within 24 hours compared to the age of gestation less than 37 weeks (OR 1.2, 95% CI: 1.2–11.3, and *P* < 0.05).

### 3.3. Risk Perceptions and Barriers of the Hepatitis B Birth Dose

The characteristics of participants who attended in-depth interviews and FGDs are presented in Table 4.

A sample of 30 parents/caregivers of the 292 participants, whose children did not receive the timely hepatitis B birth dose, participated in an in-depth interview or the FGDs. We identified several common opinions where it suggested that participants knew the risks of hepatitis B and ways of transmission, with a majority of parents/caregivers being aware that anybody could get HBV, which included their children. However, they seldom mentioned that HBV could be transmitted through MTCT, sexual contact, and blood.

  “I think the children's immune system is weak; so, they are susceptible to hepatitis B” (female, FGDs 01-4 and male, a participant in FGDs 03-1).
  “Hepatitis B is spread via the respiratory; so, we kept our distance” (female, a participant in FGDs 05-3).
  “Transmitted by contact with an infected person” (female, in-depth interviews 05).

Some participants in the FGDs had some opinions about issues relating to the birth dose. These included the side effects after vaccination (fever, shock, or death) in premature infants.

One woman said, “If children are premature, they should not get the hepatitis B birth dose vaccine within 24 hours” (female, a participant in FDGs 01-1).

One man in the FGDs said, “After getting a vaccination, children can get a fever, convulsions, or die” (male, a participant in FGDs 03-3).

## 4. Discussion

The effect of the HBV birth dose is found in some surveys in Vietnam, whereby the rate of HBsAg positive in children vaccinated with 4 doses of the HBV vaccine including a birth dose is lower than those who did not receive the birth dose or were not vaccinated (1.75%, 2.98%, and 3.47%, respectively) [12]. Within the population of 12–59-month-old children in Dist. 2 Hospital, our study showed that the prevalence of the hepatitis B birth dose within 24 hours was only 71.6%, and that was lower than the rate of three doses of the pentavalent vaccine (94.9%). The results may show that children who received a timely hepatitis B birth dose were very likely to complete the full pentavalent series, which makes it even more important to start vaccination with a timely hepatitis B birth dose. The rate of birth dose in our study was higher than the reported number with nearly 55% after the Vietnamese media published the noted information in 2013 [14] and almost 70% in 2015-2016 in a national survey reported in Vietnam [15]. Our previous study in Ho Chi Minh City also correlated with the birth dose coverage in 2014-2015 (45.2%) [16]. Recently, a study in Vietnam showed that only 62.8% of children received the HBV birth dose [12]. Our results were also similar to a research in the Philippines (72% in 2017 and 65% in 2018). However, it was far lower than that reported in Thailand (99% in 2017 and 97% in 2018) and China (99% in 2017 and 2018) [17]. The low birth dose rate in our results could be explained by associated factors such as 5.1% of children had a gestation period of less than 37 weeks, and there was a noted resistance in receiving the hepatitis B birth dose, which was found in the in-depth interviews and FGDs. Besides, noting the adverse events following the immunization (AEFI) in 2013 in Vietnam, healthcare workers were reluctant to vaccinate, especially after the Ministry of Health issued a circular limiting the number of children who would fit the criteria for receipt of the vaccine [18]. However, we did not find any difference in the rates of the birth dose in children before and after 2013. The results may be affected by the use of a small sample. This is also in line with our earlier observations, which indicated that many mothers had fears of adverse events relating to the vaccination [16]. According to WHO, these were not listed as valid reasons to not receive the birth dose vaccination. The WHO recommended that all infants (including low birth weight and premature infants) should receive their first dose of the hepatitis B vaccine as soon as possible after birth, ideally within 24 hours [19]. Therefore, the management of the child should include timely HBV vaccination, which is based on WHO recommendations. In-depth interviews and FGDs recorded parents' opinions about risk perception and barriers to the hepatitis B birth dose vaccine. This approach is recommended for encouraging a willingness to share information among parents/caregivers with similar experiences and to discuss complex or sensitive problems. It has been recommended that 30–40 participants are needed to have a sufficient breadth of input to detect a theme or concept [20]. Despite previous media campaigns focused on vaccinations for children, our study showed a lingering number of misconceptions about HBV risks, the modes of transmission, and barriers to the hepatitis B vaccine birth dose. Similarly, such misconceptions have also been found in the studies in Ho Chi Minh City [21–24]. In another study in Vietnamese Americans, 38.7% of them believed that people could prevent HBV by avoiding HBV positive individuals or improving hygiene, diet, and exercise [25]. They all reflected an inadequate understanding of transmission. Barriers for timely hepatitis B birth dose in our study showed that the participants had concerns relating to the side effects after vaccination, and the specific concerns about children who were premature or having a low birth weight. This finding was also reported in previous studies in Vietnam [12, 26]. Also, the new regulation, Circular 12 of Vietnam Ministry of Health, has been issued, which helps healthcare workers delivering immunization and has contributed to placing special attention on the safety of HBV injections and AEFI. Recommendations showed some limitations, such as no more than 50 children per each immunization session, and this may affect Ho Chi Minh City as it is one of the areas with a high-target population [18]. Therefore, the immunization service provision should be optimized through the application of the WHO recommendations and organization of an adequate number of sessions. A study in Vietnam in 2008 also found that similar barriers for a timely hepatitis B birth dose included conflicting guidelines at hospitals and low birth weight [27]. In other research, both Vietnamese and Korean Americans showed that participants had many difficulties in paying for HBV immunizations [28]. Australian midwives relied on their clients in vaccine selection, and along with a lack of explanation of vaccination timings in preventing perinatal transmission, they contributed as a barrier to the receiving of the birth dose [29]. Similar concerns were noted in midwives in rural Indonesia who reported concerns about adverse reactions, contributed to the rates of the birth dose [30]. According to the WHO, these factors were not included in the list of contraindications for hepatitis B vaccination [31]. We found that all barriers were derived from a lack of understanding by parents or healthcare providers. Therefore, the health promotion efforts need to concentrate on removing all false contraindications (including low birth weight and premature birth) to help parents and healthcare workers have a thorough insight into the actual risks of vaccination and reduce the misconceptions relating to the vaccination of children who have low birth weight or gestation periods of less than 37 weeks.

## 5. Limitations

Some limitations of our study include the small percentage of the study population that agreed to participate and only children with vaccination cards were eligible. Additionally, events over a long time interval are being asked about, and they may happen recall bias. Also, the study has not collected variables including antenatal visits, BCG vaccine, and vaccination barriers among healthcare workers. Future studies should be concentrated on these issues. Although not generalizable, it provided insights into the local sociocultural context of parents to design appropriate intervention and an outreach plan.

## 6. Conclusion

The rate of hepatitis B birth dose was low, which resulted from associated factors such as premature birth, likely to be linked with false contraindications and beliefs that, potentially, the 2013 incident is still fresh in people's minds. Therefore, strategies to implement policies around the birth dose HBV should be in line with current WHO recommendations and strategies to modify current beliefs about vaccination.

## Figures and Tables

**Table 1:** Baseline characteristics of children and parent/guardian respondents (*N* = 292).

	*N* (%)
*Baseline characteristics of parents/caregivers*	
Gender (female)	259 (88.7)
Education	
Primary and secondary school	77 (26.4)
>High school	215 (73.6)
Occupation	
Government officer/staff	122 (41.8)
Housewife	104 (35.6)
Seller/retail	44 (15.1)
Others	22 (7.5)
Age (years) (M ± SD)	32.7 ± 6.8
Information about hepatitis B vaccination (yes)	232 (79.5)

*Baseline characteristics of children*	
Gender (male)	160 (54.8)
Age (months)	30.3 ± 13.9
Period of delivery	
<2013	32 (10.9)
≥2013	260 (89.1)
Birth order of child in the family	
1^st^	109 (37.3)
2^nd^	159 (54.5)
≥3^rd^	24 (8.2)
Place of birth	
Dist. 2 Hospital	142 (48.6)
Tu Du Hospital	105 (36.0)
Others	45 (15.4)
Moderate birth weight (>2,500 g)	281 (96.9)
Age of gestation (<37 weeks)	15 (5.1)
Caesarean	107 (36.6)

**Table 2:** Hepatitis B vaccination status of children (*N* = 292).

Hepatitis B vaccination status	*N* (%)
Birth dose within 24 hours	209 (71.6)
Three doses of pentavalent vaccine on time (2, 3, and 4 months)	277 (94.9)
Complete and timely HepB vaccine (birth dose within 24 hours and three doses of pentavalent on time)	208 (71.2)

**Table 3:** The association between baseline characteristics of participants and the birth dose of hepatitis B vaccine (*N* = 292).

	Hepatitis B vaccine birth dose		*P* value	AOR	95% CI	*P* value
	Yes (209) No. (%)	No. (83) No. (%)				
*Baseline characteristics of parents*						
Gender (female)	182 (87.1)	77 (92.8)	0.166^*∗*^	—	—	—
Education						
Primary and secondary school	52 (24.9)	25 (30.1)	0.359^*∗*^	—	—	—
≥ High school	157 (75.1)	58 (69.9)		—	—	—
Occupation						
Seller/retail	37 (17.7)	7 (8.4)	0.187^*∗*^	—	—	—
Housewife	69 (33.0)	35 (42.2)		—	—	—
Government officer/staff	87 (41.6)	35 (42.2)		—	—	—
Others	16 (7.7)	6 (7.2)		—	—	—
Age (years) (M ± SD)	32.9 (±0.46)	32.3 (±0.79)	0.464^*∗∗∗*^	—	—	—
Hepatitis B vaccine information (yes)	174 (83.3)	58 (69.9)	**0.011** ^*∗*^	0.8	0.6–1.2	0.234^#^

*Baseline characteristics of children*						
Gender (male)	115 (55.0)	45 (54.2)	0.901^*∗*^	—	—	—
Age (months)	30.8 ± 0.9	28.9 ± 1.5	0.277^*∗∗∗*^	—	—	—
Period of delivery						
<2013	27 (12.9)	5 (6.0)	0.089^*∗*^	—	—	—
≥2013	182 (87.1)	78 (94.0)		—	—	—
Birth order of child in the family						
1^st^	77 (36.8)	32 (38.6)		—	—	—
2^nd^	116 (55.5)	43 (51.8)	0.787^*∗*^	—	—	—
≥3^rd^	16 (7.7)	8 (9.6)		—	—	—
Place of birth					—	
District 2 Hospital	101 (71.1)	41 (28.9)	0.959^*∗*^	—	—	—
Tu Du Hospital	75 (71.4)	30 (28.6)		—	—	—
Others	32 (72.7)	12 (27.3)		—	—	—
Low birth weight (<2,500 g)	7 (3.3)	2 (2.4)	0.504^*∗∗*^	—	—	—
Age of gestation (≥37 weeks)	206 (98.6)	71 (85.5)	**<0.001** ^*∗∗*^	1.2	1.2–11.3	**<0.05** ^**#**^
Caesarean (yes)	78 (37.3)	29 (34.9)	0.703^*∗∗*^	—	—	—

^*∗*^Chi-square, ^*∗∗*^Fisher's exact, ^*∗∗∗*^*t*-tests used to compare with and without the birth dose of hepatitis B vaccine groups. ^#^*Z*-test was used for coefficients of the logistic regression model, “—” if the multivariate analysis is not available.

**Table 4:** Demographic characteristics of participants in-depth interviews and FGDs (*n* = 30).

	FGDs no. (%)	In-depth interviews no. (%)	Total no. (%)
Amount	15	15	30
Gender			
Female	10 (66.7)	12 (80.0)	22 (73.3)
Male	5 (33.3)	3 (20.0)	8 (26.7)
Education			
Primary and secondary school	6 (40.0)	5 (33.3)	11 (36.7)
High school	9 (60.0)	10 (66.7)	19 (63.3)
Occupation			
Government officer/staff	5 (33.3)	6 (40.0)	11 (36.7)
Housewife	9 (60.0)	4 (26.6)	13 (43.3)
Seller	1 (6.7)	5 (33.4)	6 (20)
Age (years) M ± SD	28.5 ± 1.7	29.3 ± 4.8	28.9 ± 3.6

## Data Availability

The data used to support this study are available from the first author upon request.
